# Preoperative systemic inflammation score (SIS) is superior to neutrophil to lymphocyte ratio (NLR) as a predicting indicator in patients with esophageal squamous cell carcinoma

**DOI:** 10.1186/s12885-019-5940-6

**Published:** 2019-07-22

**Authors:** Xiaobin Fu, Tingting Li, Yaqing Dai, Jiancheng Li

**Affiliations:** 1 Department of Radiation Oncology, The Second Affiliated Hospital of Fujian Medical University, Quanzhou, 362000 Fujian China; 2 Department of Radiation Oncology, Fujian Medical University Cancer Hospital & Fujian Cancer Hospital, 420 Fuma Road, Jin’an District, Fuzhou, 350014 Fujian China

**Keywords:** Esophageal squamous cell carcinoma, Systemic inflammation score, Neutrophil to lymphocyte ratio, Prognosis

## Abstract

**Background:**

The aim of this study was to assess the prognostic significance of preoperative systemic inflammation score (SIS) on patients with esophageal squamous cell carcinoma (ESCC).

**Methods:**

A total of 357 ESCC patients who accepted radical esophagectomy between January 2008 and December 2009 at our institution were recruited in the analysis. The cut-off finder application was used to calculate the optimal cutoff values. The Chi-squared test or Fisher’s exact test were used to analyze categorical variables. Overall survival (OS) was calculated using the Kaplan-Meier method and the log-rank test. Multivariate analysis was calculated using Cox regression analysis model. A model combining SIS was created and its performance was evaluated using the Akaike information criterion (AIC) and concordance index (C-index).

**Results:**

The median follow-up time was 58 months (range, 1–84 months). The 5-year OS rate was 50% (95% CI, 49.94–50.06%). The optimal cut-off values for preoperative neutrophil to lymphocyte ratio (NLR), lymphocyte-to-monocyte ratio (LMR) and serum albumin (Alb) were 2.27, 3.79 and 36.55, respectively. Univariate analyses revealed that gender (*P* = 0.047), T stage (*P* < 0.001), N stage (*P* < 0.001), vascular invasion (*P* < 0.001), tumor location (*P* = 0.018), tumor length(*P* < 0.001), NLR (*P* = 0.006), LMR (*P* = 0.007), serum Alb (*P* = 0.001), and SIS (*P* < 0.001) were significantly associated with OS. Independent prognostic factors for OS were T stage, N stage, tumor location, tumor length, and SIS. However, NLR was not an independent prognostic factor in multivariate analysis. The model combining SIS had smaller AIC and higher C-index compared to the model without SIS, which suggesting that the adding the SIS to the multivariate model increasing the predictive accuracy of the OS in the ESCC patients treated with radical esophagectomy and 3-field lymphadenectomy (3-FL).

**Conclusions:**

SIS may treat as a novel prognostic factor than NLR for ESCC patients who underwent radical esophagectomy and 3-FL. However, Larger-scale studies are needed to validate these findings.

**Electronic supplementary material:**

The online version of this article (10.1186/s12885-019-5940-6) contains supplementary material, which is available to authorized users.

## Background

There are huge differences between the United States (US) and China in the incidence and pathologic type of esophageal carcinoma (EC). Unlike the US, EC is one of the prevalent malignant carcinoma in China with high incidence and mortality and squamous cell carcinoma is the main pathological type which accounted for 90% of EC. The crude incidence rate of esophageal carcinoma in 2014 was 12.17/100,000, which represented 18.85% of all estimated cancer cases [1, 2]. With the significant development in treatment methods for esophageal squamous cell carcinoma (ESCC), the average survival rate increased by 2.9% per calendar period from 2003 to 05 to 2012–15. However, the prognosis of ESCC remained poor and the 5-year survival rate was merely 30.2% [3].

Previous in vitro and in vivo studies revealed that the degree of systemic inflammatory response significantly affected the outcomes in various solid carcinomas including the stomach, kidney, and esophageal carcinoma by increasing the probability for primary tumor invasion, distant metastasis, and immune tolerance [4–7]. Based on the relationship between inflammatory response and overall survival, the inflammation-based factors including Glasgow prognostic score, neutrophil to lymphocyte ratio (NLR), platelet to lymphocyte ratio (PLR), and prognostic nutritional index (PNI) have been showed to have prognostic value in cancer patients [8–10] .

Among the inflammation-based factors mentioned above, NLR was widely reported as a timesaving, economical, repeatable and routine inflammation-based prognostic indicators were widely used to monitor the degree of systemic inflammatory response for various solid carcinoma and predict the patient prognosis in the recent studies [11–14]. However, there is no widely accepted optimal cutoff value for NLR and no established scoring system that combines the inflammation indicators to predict the prognosis of cancer patients. Recently, the systemic inflammation score (SIS), combined the pretreatment albumin levels and lymphocyte to monocyte (LMR), was reported as a novel prognostic indicator for gastric carcinoma and clear cell renal cell carcinoma [15, 16]. However, the studies about the predictive value of systemic inflammation score (SIS) in esophageal carcinoma are few, and no studies regarding the prognostic value of preoperative SIS compared with NLR in ESCC treated with radical esophagectomy and 3-field lymphadenectomy (3-FL).

Thus, the aim of this study was to assess the predictive value of preoperative SIS in ESCC patients treated with the radical esophagectomy and 3-FL.

## Methods

### Patients

This study was approved by the Ethics Committee of the Fujian Provincial Cancer hospital (NO. KT2018–014-01). 357 consecutive ESCC cases who meet the following criteria were recruited in this retrospective study, (a) the Karnofsky score ≥ 80 points, (b) pathologically confirmed as ESCC, (c) the patients received radical esophagectomy and 3-FL with at least 15 lymph nodes resected, (e) without a history of malignant disease, and (f) the patients received blood routine test and biochemical examination 7 days prior to surgery.

### Surgical strategy

All the ESCC patients included in the study received radical esophagectomy by total thoracotomy on the left side and intrathoracic gastric reconstruction. All the patents underwent the 3-field lymphadenectomy (neck, mediastinum and abdomen) which including the supraclavicular, paravaryngeal, paratracheal, paratronchial, paraesophageal, subcarinal, diaphragmatic, and paracardiac lymph nodes, as well as lymph nodes located along lesser gastric curvature, left gastric artery lymph nodes.

### Radiation and chemotherapy

In this study, 214 ESCC patients received intensity-modulated radiation therapy (IMRT) and chemotherapy. The cervical, thoracic and abdominal parts were fixed using plastic sheet or vacuum pad. Imaging data were collected from prior computed tomography (CT) simulation scan and transmitted to radiation therapy treatment planning system (Pinnacle, Philips Radiation Oncology System, USA) to delineate the tumor area and the organs at risk according to the criteria of tumor sketching of the National Comprehensive Cancer Network (NCCN). Additional parameters included prescribed dose of 50–66 Gy, median dose of 60 Gy, Bi-lung V20 ≤ 20%, an average bi-lung dose of ≤20 Gy, a bi-lung V5 of < 50%, a heart V30 of ≤30%, and a maximum dose to the spinal cord of < 45 Gy. The chemotherapy regimens used in ESCC patients was as followed, docetaxel 135–175 mg/m2 D1 + cisplatin 80 mg/m2 D2.

### Definition of the NLR, LMR and SIS

Data of the pretreatment absolute blood cell counts were collected from the previous blood routine test records in Fujian Provincial Cancer Hospital. The NLR was calculated by dividing the absolute neutrophil count by the absolute lymphocyte count. The LMR was defined as the absolute lymphocyte count divided by the monocyte count. SIS defined based on the combination of the preoperative serum Alb and LMR was as followed, a) patients with both increased LMR and increased serum Alb were defined as a score of 0, b) patients with either increased LMR or increased serum Alb were defined as a score of 1, c) patients with both decreased serum Alb and decrease LMR were defined as a score of 2 [15, 16].

### Pathological staging

All the ESCC patients were performed the pathological TNM staging based on the pathological diagnosis by the experienced oncologist according to the 8th edition TNM stage issued by American Joint Committee on Cancer (AJCC). For the T1a ESCC with tumor cell G1, the patients were classified as stage Ia. For the T1a ESCC with tumor G2/3, T2 ESCC with tumor cell G1 and T1b ESCC, the patients were classified as stage Ib. For the T2 ESCC with tumor cell G2/3, the T3 ESCC with tumor cell G1 and T3 ESCC with tumor cell G2/3 located in the lower third, the patients were classified as stage IIa. For the T3ESCC with tumor cell G2/3 located in the middle and upper third and the T1 N1, the patients were classified as stage IIb. For the T1 N1 and T2 N1 ESCC, the patients were classified as stage IIIa. For the T2 N2, T3 N1–2 and T4aN0–1 ESCC, the patients were classified as stage IIIb. For the T4bN0–3 and T1-4 N3 ESCC, the patients were classified as Iva.

### Follow-up

A regular follow-up examination was conducted every 3 months the first year, every 6 months the next 2 years, and once per year thereafter. The routine examination included physical examination, routine blood test, biochemical examination, the thoracic and upper abdominal CT scan, barium meal radiograph et.al. December 2014 was the last censoring date for evaluating survival time. Survival time was defined as the interval between the date of surgery to the death or last follow-up.

### Statistical analysis

All recorded data were calculated by SPSS (version 19.0, SPSS Inc., Chicago, IL, USA) and the statistical software “R” (version 2.11.1, the R Foundation for statistical computing). The cutoff finder application was performed to calculate the optimal cutoff value. The Chi-squared test or Fisher’s exact test was used to compare the differences of pathological stage factors in the patients grouped by NLR and SIS. The survival rate was calculated using the Kaplan-Meier method, and a log-rank test was used to assess the survival differences between groups. Cox proportional hazards regression analysis was performed to identify independent factors that were correlated with the patients’ overall survival. The Akaike information criterion (AIC) was used to identify a superior multivariate prediction model. The predictive accuracy of the two models was also identified by the concordance index (C-index), which ranged from 0 to 1. The corresponding interval (CI) was calculated by bootstrapping and *p*-value of C-index was calculated according to assume asymptotic normality. The larger C-index value revealed a better predictive accuracy. All tests were two-sided, and a *P* value< 0.05 was considered statistically significant.

## Result

### Patients’ characteristics

A total of 357 patients (279 males and 78 females) meet the inclusion criteria were enrolled (Table 1). The median age was 57 years (range, 34 to 77y). The patients for tumor located in upper third (UE), middle third (ME), and lower third (LE) were 60 (16.8%), 260 (72.8%), and 37 (10.4%), respectively. The tumor cell G1, G2, and G3 were 54 (15.1%), 258(72.3%), and 45 (12.6%), respectively. The patients for stage Ib, IIa, IIb, IIIa, IIIb, IVa were 29(8.1%), 71(19.8%), 52(14.6%), 23(6.4%), 126(35.2%), and 56(15.7%) respectively. The association of the NLR and SIS are showed in the Table 2. Our study showed that high NLR was associated with male sex (*P* = 0.003), tumor length (< 0.001), and T stage (*P* = 0.002). The high SIS was associated with increased tumor length (*P* = 0.004) (Additional file 1).

**Table 1:** Patients’ characteristics for 357 ESCC patients

Characteristics	Number of patients (%)
Gender	
Male	279 (78.2)
Female	78 (21.8)
Age (years)	
≤ 65	276 (77.3)
> 65	81 (22.7)
Vascular invasion	
Yes	312 (87.4)
No	45 (12.6)
Tumor cell differentiation	
G1	54 (15.1)
G2	258 (72.3)
G3	45 (12.6)
Tumor location	
Upper third	60 (16.8)
Middle third	260 (72.8)
Lower third	37 (10.4)
T stage	
T1	40 (11.2)
T2	71 (19.9)
T3	209 (58.5)
T4	37 (10.4)
N stage	
N0	155 (43.4)
N1–3	202 (56.6)
Pathological stage	
I	29 (8.1)
II	123 (34.5)
III	149 (41.7)
IVa	56 (15.7)
Adjuvant therapy	
Yes	214 (60)
No	143 (40)

**Table 2:** ESCC Patients’ clinicopathological characteristics according to NLR and SIS

characteristics	NLR			SIS groups			
	NLR ≤ 2.27	NLR > 2.27	P	SIS = 0	SIS = 1	SIS = 2	P
	*N* = 228	*N* = 129		*N* = 119	*N* = 148	*N* = 90	
Gender			0.003				< 0.001
Male	167	112		71	128	80	
Female	61	17		48	20	10	
Age			0.874				0.087
≤ 65 years old	177	99		95	119	62	
>65 years old	51	30		24	29	28	
Vascular Invasion^a^			0.453				0.944
No	197	115		103	130	79	
Yes	31	14		16	18	11	
Tumor cell differentiation^a^			0.561				0.412
G1	31	23		14	21	15	
G2	168	90		90	109	18	
G3	29	16		15	18	12	
Tumor location^a^			0.728				0.324
Upper	41	16		21	26	7	
Middle	164	96		91	105	17	
Lower	23	14		7	64	13	
Tumor length			< 0.001				0.004
≤ 5 cm	167	69		91	98	49	
> 5 cm	61	60		28	50	41	
T stage			0.002				0.102
T1	33	7		18	14	8	
T2	53	18		26	34	11	
T3	124	85		60	89	60	
T4	18	19		15	11	11	
N stage			0.266				0.161
N0	104	51		58	65	32	
N1–3	124	78		61	83	58	
Stage			0.109				0.117
I	24	5		14	12	3	
II	81	24		43	51	29	
III	90	59		49	61	39	
IVa	33	23		13	24	19	

^a^ While *N* ≥ 40 and 1 ≤ theoretical frequency(T)<5, the Fisher exact test was used to compare the influencing factors

### ROC curve for prediction

**Fig. 1:**
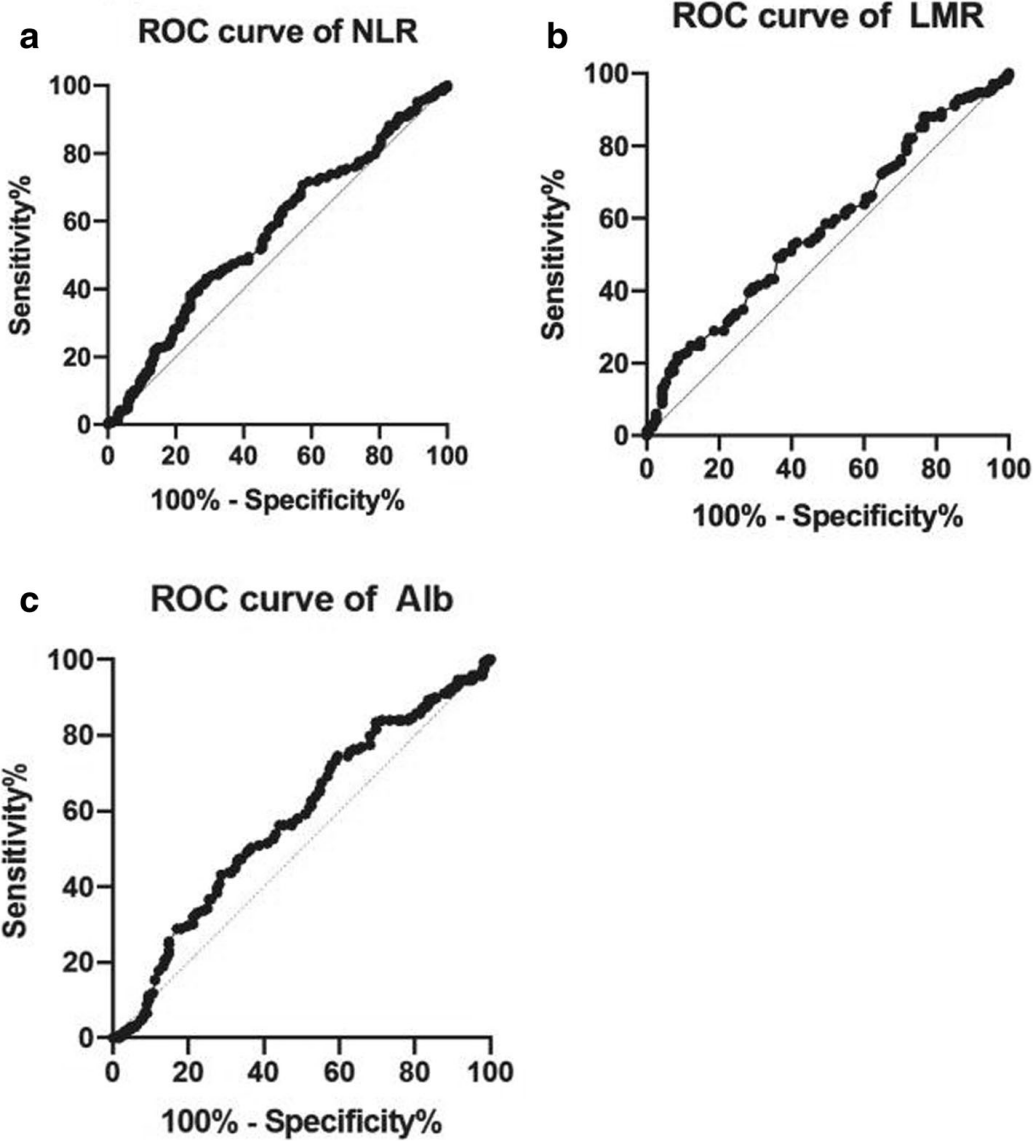
ROC curve of NLR (**a**), LMR (**b**), and Alb (**c**) for predicting the ESCC patients’ prognosis

For NLR, the area under the curve (AUC) was 0.566 (95% CI 0.507–0.626). The optimal cutoff value was 2.27. The sensitivity and specificity were 0.431 and 0.71. For LMR, the area under the curve (AUC) was 0.576 (95% CI 0.517–0.636). The optimal cutoff value was 3.94. The sensitivity and specificity were 0.491 and 0.638. For serum Alb, the AUC was 0.578 (95% CI 0.518–0.637). The optimal cutoff value, sensitivity, and specificity were 36.55, 0.746, and 0.404. ROC curves of NLR, LMR, and Alb are shown in the Fig. 1a, b, and c. SIS defined based on the combination of the preoperative serum Alb and LMR was as followed, a) patients with both LMR > 3.94 and serum Alb> 36.55 were defined as a score of 0, b) patients with either LMR > 3.94 or serum Alb> 36.55 were defined as a score of 1, c) patients with both serum Alb≤36.55 and LMR ≤ 3.94 were defined as a score of 2.

### Survival for the whole cohort and prognostic impact of the SIS and NLR

**Fig. 2:**
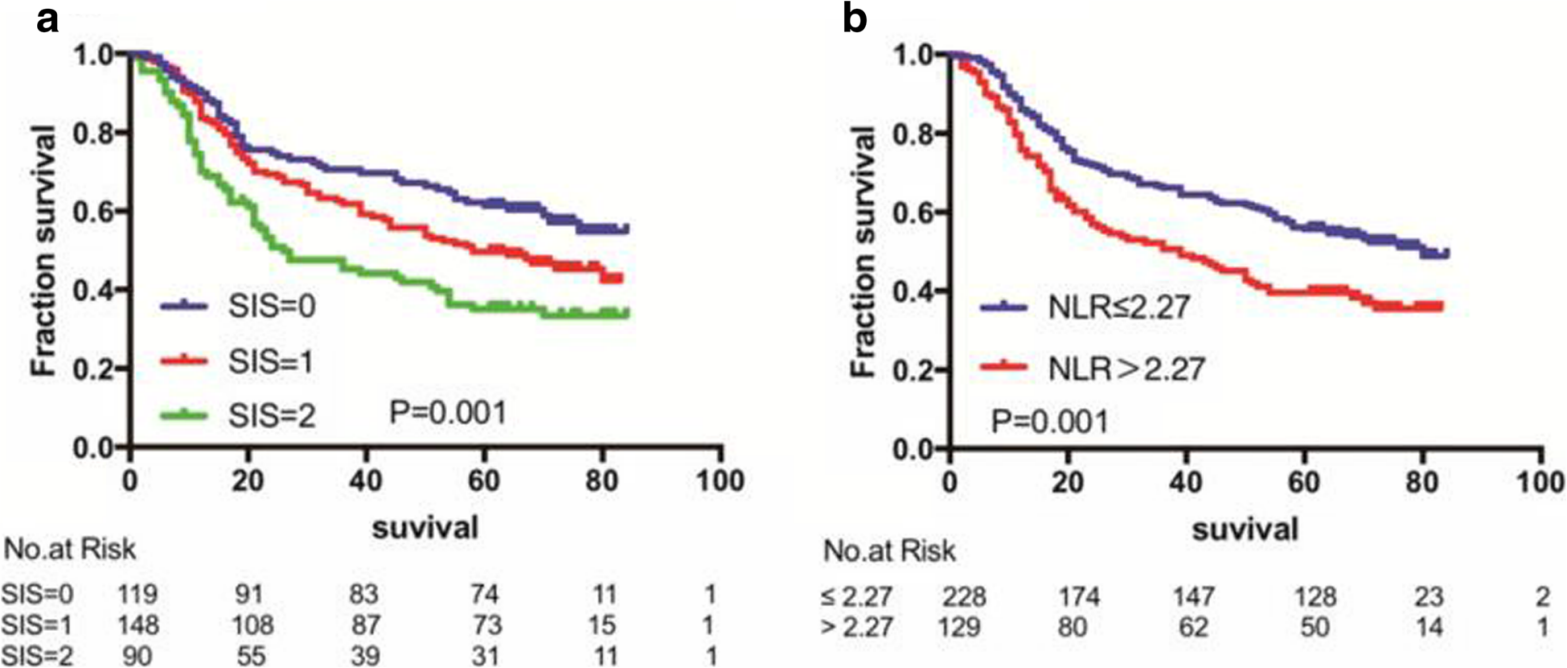
Kaplan-Meier analysis for overall survival of 357 ESCC patients according to systemic inflammation score = 0, =1 versus =2 (**a**) and neutrophil to lymphocyte ratio ≤ 2.27 versus>2.27 (**b**)

The median follow-up time was 58 months (ranging: 1–84 months). Among the 357 ESCC cases, the 1-year, 3-year, and 5-year survival rate were 82% (95%CI, 81.96%-82.04), 61% (95%CI, 60.96%-61.04), and 50% (95%CI, 49.94%-50.06), respectively. In our cohort, for NLR ≤2.27 (*n* = 228) and NLR > 2.27 (*n* = 129), the 5-year survival rate were 56% (95%CI, 59.94–56.06%) and 40% (95%CI, 39.92–40.08%), respectively. For SIS = 0 (*n* = 64), SIS = 1 (*n* = 60), and SIS = 2 (*n* = 20), the 5-year survival rate were 61% (95%CI, 60.92–61.08%), 50% (95%CI, 49.92–50.08%) and, 35% (95%CI, 34.9–35.1%) respectively. Kaplan–Meier analysis demonstrated that the association of SIS and NLR with OS. High SIS and low NLR were associated with inferior OS (for the SIS, *P* = 0.001; for the NLR, *P* = 0.006). Kaplan–Meier curves of OS based on pretreatment SIS and NLR are shown in Fig. 2a and b.

### Prognostic factors affecting OS in the whole cohort

Univariate analysis demonstrated that gender (*P* = 0.047), T stage (*P* < 0.001), N stage (*P* < 0.001), vascular invasion (*P* < 0.001), tumor location(*P* = 0.018), adjuvant CRT(*P* = 0.036), tumor length(*P* < 0.001), NLR (*P* = 0.006), and SIS (P < 0.001) were significant associated with OS of 357 ESCC patients. Multivariate analysis showed that T stage (*P* = 0.008), N stage (*P* < 0.001), tumor location (*P* = 0.025), tumor length (*P* = 0.002), and SIS (*P* = 0.029) were independent prognostic factors (Table 3).

**Table 3:** Univariate and multivariate analysis of 5-year overall survival

Variables	Univariate		Multivariate		
	5-year OS	*P* value (log-rank)	Hazard ratio	95%CI	*P* value
Gender		0.047			
Male	279				
Female	78				
Age (years)		0.159			
≤ 65	276				
> 65	81				
T stage		< 0.001	1.323	1.12–1.53	0.008
T1	40				
T2	71				
T3	209				
T4	37				
N stage		< 0.001	1.525	1.4–1.65	< 0.001
N0	155				
N1	87				
N2	69				
N3	46				
Vascular invasion		<0.001			
No	45				
Yes	312				
Tumor cell		0.61			
G1	54				
G2	258				
G2	45				
Tumor location		0.018	1.361	1.09–1.63	0.025
Upper third	60				
Middle third	260				
Lower third	37				
Adjuvant CRT		0.036			
Yes	214				
No	143				
Tumor length		< 0.001	1.609	1.31–1.9	0.002
≤ 5 cm	236				
> 5 cm	157				
SIS		< 0.001	1.24	1.05–1.43	0.029
0	119				
1	148				
2	90				
NLR		0.006			
≤ 2.27	228				
>2.27	129				

### Comparisons between the two multivariate models

The comparisons of multivariate models 1 (gender, age, T stage, N stage, vascular invasion, tumor cell differentiation, pathological stage, tumor length, and tumor location) and multivariate model 2 (gender, age, T stage, N stage, vascular invasion, tumor cell differentiation, pathological stage, tumor location, tumor length and SIS) assessed by the AIC, C-index, and likelihood ratio χ2 score (Table 4). The AIC values in the multivariate model 1 and 2 were 1999.66 and 1999.53. The AIC value was smaller in the model 2, suggesting that combining SIS to the multivariate model enabled a superior prediction model for OS. Moreover, the C-index value in the multivariate model 1 and 2 were 0.715 (0.672–0.759) and 0.718 (0.675–0.762). The C-index increased slightly in the model 2, which suggesting that the adding the SIS to the multivariate model increasing the predictive accuracy of the OS in the ESCC patients treated with radical esophagectomy and 3-FL. However, the C-index of the model 2 was not significant differences compared to the model 1 (*P* = 0.91).

**Table 4:** Comparison of different prognostic models on 357 ESCC patients

	Concordance Indices		AIC	Likelihood Ratio X^2^
	C-index	Bootstrap 95% CI		
Model 1	0.715	0.672–0.759	1999.66	93.013
Model 2	0.718	0.675–0.762	1999.53	95.146

## Discussion

Pretreatment prognostic factors including TNM staging, tumor grade, tumor location, and tumor burden might not comprehensively predict the prognosis of patients with ESCC. Accumulating studies have revealed that it is not just the influencing factors mentioned above affecting the ESCC patients’ prognostic. Moreover, inflammation-based factors and immunonutritional indicators such as the pretreatment NLR, PLR, LMR, and PNI have been evaluated as possible prognostic factors for esophageal carcinoma. In addition to the inflammation indicators mentioned above, SIS, as a superior inflammation-associated prognostic score, was established based on the combination of the preoperative serum Alb and LMR, had found strongly prognostic value in our study. To our best knowledge, no studies regarding the prognostic value of preoperative SIS compared with NLR in ESCC. All the ESCC patients in our study underwent radical esophagectomy and 3-FL which eliminated the influence of surgical mode and pathological type on prognosis. We had shown that the ESCC patients with NLR ≤ 2.27, LMR > 3.94, Alb> 36.55 and low SIS had better OS. Moreover, the SIS was the independent prognostic factors for overall survival in our study. However, the NLR, was not independent prognostic factors in multivariate analysis. The ESCC patients with low SIS had a significantly greater 5-year OS rate than those with high SIS (*P* < 0.001). SIS may treat as a novel prognostic factor than NLR for patients with ESCC who underwent radical esophagectomy and 3-FL.

Nowadays, a superior inflammation marker, the SIS, was reported to have the value in predicting the outcomes in the solid tumors such as gastric cancer, non-small cell lung cancer, cervical carcinoma et.al [15, 17, 18]. However, the studies focused on the relationship between the SIS and the ESCC patients’ outcome were rare. Jian-Xian Lin et.al [15], studied 1786 gastric cancer accepted the curative resection and revealed that the SIS was the independent prognostic factor in the gastric cancer patients. In that study, the SIS was established based on the combination the pretreatment serum Alb and LMR. Patient with low score (both elevated Alb and LMR) had a significant survival benefit compared with the patients with high score (both decreased Alb and LMR). In another study by Masaki Tomita et.al [17], who studied 341 non-small cell lung cancer and showed that SIS was a novel independent prognostic factors in predicting the non-small cell lung cancer patients’ OS. In that study, the SIS was based on the combination the pretreatment serum C-reactive protein and Alb and showed that the patients with high SIS (C-reactive protein> 10 ng/L and Alb< 35 g/L) had adverse impact on the patients’ overall survival. Ru-ru Zheng et.al [18] performed the multivariate model analysis on 795 resectable cervical cancer showing that the SIS, combined the pretreatment serum Alb and PLR, was the independent prognostic factor. The patients with high SIS (Alb< 43.65 g/L and PLR ≥ 128.3) had a worse 5-year OS and 5-year disease free survival (DFS) than the patients with low SIS (Alb≥43.65 g/L and PLR < 128.3). In addition to the solid cancer mentioned above, some studies also revealed the relationship between the LMR and outcomes in the esophageal carcinoma. Lihui Han et.al [19], studied the 206 ESCC patients underwent esophagectomy and found that the patients with low SIS (pretreatment serum Alb≥43.1 g/L and LMR ≥ 2.9) had a better 5-year disease free survival and overall survival than the patients with high SIS (pretreatment serum Alb< 43.1 g/L and LMR < 2.9). Similar to this study, the optimal cutoff value of Alb and LMR in our study were 36.55 g/L and 3.94. The patients with SIS = 0 (both Alb> 36.55 g/L and LMR > 3.94) had a better 5-year overall survival than the patients with SIS = 1(either Alb> 36.55 g/L or LMR > 3.94) and SIS = 2(both Alb≤36.55 g/L and LMR < 3.94) and the difference had significantly statistical difference. Moreover, the SIS was the independent prognostic factor by performing the multivariable code model analysis.

The underlying biological mechanism of SIS in impacting the patients’ prognosis might be determined by serum Alb and LMR. Some in vivo and in vitro studies verified that the low LMR and decreased serum albumin were significant correction with the poor prognosis of the cancer patients [20–22]. Hypoalbuminemia represents the poor nutritional status and increased inflammatory degree which potentially exerting negative impact on the ESCC patients’ survival [23] . Moreover, hypoalbuminemia also decreases the agents such as cholesterol, fatty acid, et.al transporting and free oxygen radicals scavenging, which have adversely outcomes on OS [24]. LMR consists of lymphocytes and monocytes. The biological reason of LMR might be demonstrated by the function of the lymphocytes and monocytes. The lymphocytes increased the anti-tumor reaction to suppress the tumor cell proliferation, migration, and angiogenesis. Thus, the patients with lymphopenia had poor survival outcomes in cancer patients [25]. In some other studies, the neutrophils and the monocytes promoted the tumor cell proliferation and modulated the tumor microenvironment to facilitate the angiogenesis, tumor invasion and metastases, and such that the cancer patients with low monocytes have poor OS [5]. Hu et.al [22], studied 218 ESCC patients and found that the patients with LMR > 2.57 had a better 5-year OS and disease-free survival than the patients with LMR < 2.57. Similar to this study, the optimal cutoff value of LMR in our study was 3.94. The patients with LMR > 3.94 had a better 5-year OS.

Except for the SIS mentioned above, a number of studies also demonstrated that pretreatment serum NLR, PLR, and PNI also significantly affected the outcomes of the OS of the colorectal, non-small cell lung cancer, gastric cancer and ESCC patients. Hao Duan et.al [26] studied 371 ESCC patients who underwent the esophagectomy and found that the pretreatment serum NLR > 3.0 was associated with the worse cancer-specific survival and recurrence-free survival. Keisuke Kosumi et.al [27], and Hiroshi Sato et.al [28] also had the similar findings. Similar to these studies, the optimal cutoff value for NLR calculated in our study was 2.27 and the patients with NLR > 2.27 had worse OS. In addition to the pretreatment serum NLR, the pretreatment serum PLR also played a crucial role in influencing the ESCC patients’ survival. A Meta-analysis by Deng [29] showed that the elevated pretreatment serum PLR was significantly associated with the poor outcomes of patients in esophageal carcinoma. However, the NLR was not an independent prognostic factors in our study. The NLR was not an independent prognostic factor in multivariate analysis. Only the SIS was the independent prognostic factors in this study. The SIS was superior to NLR in predicting the ESCC patients’ prognosis.

This study has a few limitations. Firstly, the sample size in this study is small cause the number of ESCC patients were limited. A larger amount of data is required to verify these results. Secondly, as a retrospective study, the analytical and selection biases was inevitable. Despite the limitations mentioned above, the study was the first to reveal the prognostic value of pretreatment SIS compared with ESCC patients treated with radical esophagectomy and 3-FL. SIS, as a timesaving, economical and reliable inflammation-based factors could be taken into consideration in the ESCC patients prognostic prediction and further treatment regimen selection.

## Conclusion

Overall, SIS may treat as a novel prognostic factor than NLR for ESCC patients who underwent radical esophagectomy and 3-FL. Measurements of the SIS are economical, timesaving, and reliable in the pretreatment work-up of ESCC patients in clinical practice. It will aid the oncologists in the individual treatment regimen’ selection. Larger-scale studies are warranted to validate these findings.

## Additional file

Additional file 1: File SIS DATA information. The file included the data of the pretreatment NLR, LMR, Albumin and survival time of 357 ESCC patients. The gender, tumor location, tumor length, age, tumor cell differentiation, vascular invasion, T stage, N stage, clinical stage, NLR, LMR and albumin were divided into different subgroups according to the details list in the Tables 1 and 2. Survival time was defined as the interval between the date of surgery to the death or last follow-up. The survival group was divided into 2 subgroups (0 for survival and 1 for death). (XLSX 59 kb)

## Data Availability

The data used to support the findings of this study are included with the article and supplementary files.
